# Low cardiorespiratory fitness is associated with higher extracellular vesicle counts in obese adults

**DOI:** 10.14814/phy2.13701

**Published:** 2018-05-20

**Authors:** Natalie Z. M. Eichner, Nicole M. Gilbertson, Julian M. Gaitan, Emily M. Heiston, Luca Musante, Sabrina LaSalvia, Arthur Weltman, Uta Erdbrügger, Steven K. Malin

**Affiliations:** ^1^ Department of Kinesiology University of Virginia Charlottesville Virginia; ^2^ Division of Nephrology University of Virginia Charlottesville Virginia; ^3^ Division of Endocrinology and Metabolism University of Virginia Charlottesville Virginia; ^4^ Robert M. Berne Cardiovascular Research Center University of Virginia Charlottesville Virginia

**Keywords:** Cardiovascular disease, endothelial function, microparticles, hypertension, metabolic syndrome

## Abstract

Low cardiorespiratory fitness (CRF) is associated with cardiovascular disease (CVD) independent of obesity. Extracellular vesicles (EVs) are a novel target of CVD, however, it remains unknown if obese individuals with very poor fitness (VPF) have elevated EVs versus people with poor fitness (PF). Thus, we tested whether VPF was associated with greater EV subtypes in obese adults. Subjects with VPF (*n* = 13, VO_2_peak: 15.4 ± 0.6 mL/kg/min, BMI: 34.1 ± 1.7 kg/m^2^) and PF (*n* = 13, VO_2_peak: 25.9 ± 3.0 mL/kg/min, BMI: 32.1 ± 1.2 kg/m^2^) were compared in this cross‐sectional study. After an overnight fast, AnnexinV (AV) +/− platelet (CD31^+^/CD41^+^), leukocyte (CD45^+^/CD41^−^), and endothelial EVs (CD105^+^, CD31^+^/CD41^−^) were analyzed from fresh platelet poor plasma via imaging flow cytometry. Body fat, blood pressure (BP), and glucose tolerance (OGTT) were also tested. Body weight, BP, and circulating glucose were similar between groups, although VPF subjects were older than PF (64.0 ± 2.1 vs. 49.8 ± 4.2 year; *P *<* *0.05). People with VPF, compared with PF, had higher total AV^−^ EVs (*P = *0.04), AV ^−^ platelet EVs (CD31^+^/CD41^+^; *P *=* *0.006), and AV ^−^ endothelial EVs (CD31^+^/CD41^−^; *P *=* *0.005) independent of age and body fat. Higher AV^−^ platelet and endothelial EVs were associated with lower VO_2_peak (*r* = −0.56, *P *=* *0.006 and *r* = −0.55, *P *=* *0.005, respectively). Endothelial‐derived AV^−^/CD31^+^/CD41^−^EVs were also related to pulse pressure (*r* = 0.45, *P *=* *0.03), whereas AV^−^/CD105 was linked to postprandial glucose (*r* = 0.41, *P *=* *0.04). VPF is associated with higher AnnexinV^−^ total, endothelial, and platelet EVs in obese adults, suggesting that subtle differences in fitness may reduce type 2 diabetes and CVD risk through an EV‐related mechanism.

## Introduction

Low cardiorespiratory fitness (CRF) is characterized by reduced peak oxygen consumption (i.e., VO2peak) and is a strong independent predictor of all‐cause mortality and cardiovascular disease (CVD) (Blair et al. 1989, 1995; Wei et al. 1999; Lee et al. 2011) in normal weight and obese adults (Wei et al. 1999). In fact, obese individuals with at least moderate levels of CRF have lower rates of CVD when compared with normal weight individuals with poor fitness (Wei et al. 1999). Improvements in fitness from “unfit” to “fit” have also been shown to provide health benefit, suggesting that subtle fitness differences play a cardioprotective role in obese adults (Blair et al. 1995). However, the mechanism that accounts for such health benefit remains unclear.

Diminished fitness has traditionally been linked to CVD by biomarkers including, but not limited to: C‐reactive protein, low‐ and high‐density lipoprotein (LDL and HDL), and hyperglycemia (Mora et al. 2006). However, the American Heart Association indicates that these biomarkers explain only 40–60% of future CVD risk in both healthy and nonhealthy individuals (Heidenreich et al. 2013). Extracellular vesicles (EVs) have emerged as a potential novel biomarker and/or mediator of CVD risk (Boulanger 2010). EVs are believed to range in cell size of 100–1000 nm and have been associated with several disease states including type 2 diabetes and hypertension (Bulut et al. 2008; Nomura 2009; Amabile et al. 2010). EVs may reflect these metabolic disease states as they are products of cell activation, apoptosis, or injury from various sources, including the endothelium, platelets, and leukocytes (Arraud et al. 2014; Erdbrügger and Lannigan 2016). Indeed, recent work suggests that EVs mediate inflammatory and oxidative stress to promote endothelial and metabolic dysfunction (Jansen et al. 2013). Given that elevated fitness is associated with improved endothelial function and reductions in hyperglycemia as well as inflammation (Mills et al. 2006; Malin et al. 2013b; Lansford et al. 2016), it would be reasonable to expect fitness to relate to lower EV levels in obese individuals. Although previous literature has suggested that endothelial EVs may play an important role in predicting exercise‐mediated aerobic fitness (Van Craenenbroeck et al. 2015), no study has systematically assessed the relationship between various subtypes of EVs using advanced imaging flow cytometry (Erdbrügger et al. 2014; Erdbrügger and Lannigan 2016) with fresh plasma to identify potential clinical relevance. In particular, the current literature has focused mainly on AV^+^ EV cell types. This is a major knowledge gap since some suggest that AV^−^ EVs may carry a different or even greater clinical relevance than AV^+^‐derived EVs (Erdbrügger et al. 2014). In addition, previous literature has extensively quantified endothelial EVs (Bulut et al. 2008; Boulanger 2010; Brown et al. 2011; Jansen et al. 2013; Bruyndonckx et al. 2015) in relation to vascular health and disease, however, other subtypes such as platelet and leukocyte‐derived EVs may also regulate health via inflammatory mechanisms (Chironi et al. 2006; Dignat‐George and Boulanger 2011). Subsequently, no work exists characterizing the role of very poor aerobic fitness (VPF) compared with poor fitness (PF) on AV^−^ or AV^+^ EV subtypes to understand how subtle differences in fitness relate to less CVD risk. Thus, we tested the hypothesis that obese adults with VPF would have higher levels of total, endothelial, platelet, and leukocyte‐derived EVs when compared with people with PF. We also hypothesized that elevated EVs would correlate with increased CVD risk.

## Methods

### Subjects

In this retrospective cross‐sectional analysis of obese individuals who were part of two studies conducted in our laboratory, 26 of 39 subjects were ranked based VO_2_peak and divided into tertiles, such that only the upper and lower tertiles were included in this analysis to test effects of CRF on EVs (Table 1). The average fitness levels of the group, taking into account both sex and age, were within published guidelines for PF and VPF; however, it is important to note that not all individual subjects within each group (i.e., *n* = 3 (VPF) and 4 (PF)) met published American College of Sports Medicine guideline criteria for VPF and PF (ACSM et al. 2018). Subjects were excluded from participation if they were physically active (>60 min/week), smoking, on hormone replacement therapy, diagnosed with type 1 or 2 diabetes as well as metabolic syndrome. Subjects were also excluded if on medications known to influence insulin sensitivity (e.g., metformin, GLP‐1 agonist, etc.) or endothelial function (beta blockers, ACE inhibitors, etc.). All subjects underwent physical examination and biochemical testing to ensure safety in study participation. A resting and exercise 12‐lead EKG was also performed to assess cardiac arrthymia. All subjects provided verbal and written informed consent as approved by our Institutional Review Board.

**Table 1:** Very poor fitness (VPF) and poor fitness (PF) demographics

	VPF (range)	PF (range)	*P*‐value
*N* (M/F)	13 (0/13)	13 (4/9)	0.09
Age (years)	64.0 ± 2.1 (50–74)	49.8 ± 4.2 (19–70)	0.007
Body composition			
Body weight (kg)	91.5 ± 4.8 (62.4–121.35)	90.6 ± 3.7 (59.9–105.8)	0.87
BMI (kg/m^2^)	34.1 ± 1.7 (25.2–44.6)	32.1 ± 1.2 (25.1–39.0)	0.36
Waist circumference (cm)	105.1 ± 3.8 (89.6–130.0)	103.4 ± 3.5 (84.8–122.2)	0.73
Body fat mass (kg)	45.3 ± 3.5 (24.7–64.9)	34.6 ± 2.7 (22.5–51.2)	0.02
Body fat percent (%)	48.7 ± 1.4 (38.7–54.2)	38.1 ± 2.0 (26.7–52.0)	0.001
Fat‐free mass (kg)	46.3 ± 1.6 (37.3–59.5)	55.8 ± 2.7 (36.2–71.1)	0.006
Cardiorespiratory fitness			
VO_2_peak (L/min)	1.4 ± 0.09 (1.0–2.13)	2.3 ± 0.1 (1.4–2.9)	<0.001
VO_2_peak (mL/kg/min)	15.4 ± 0.6 (11.1–18.0)	25.9 ± 3.0 (22.7–33.1)	<0.001
Cardiovascular risk factors			
Systolic BP (mmHg)	130.1 ± 6.3 (101.0–184.0)	125.6 ± 3.0 (107.0–142.0)	0.53
Diastolic BP (mmHg)	72.8 ± 4.0 (57.0–111.0)	71.5 ± 2.4 (55.0–84.0)	0.75
MAP (mmHg)	91.9 ± 4.7 (73.5–135.3)	89.4 ± 2.3 (75.3–102.0)	0.47
Pulse pressure (mmHg)	57.4 ± 3.0 (41.3–78.5)	54.3 ± 2.7 (35.0–72.0)	0.47
AI fasting (%)	32.8 ± 3.7 (14.0–54.0)	28.7 ± 4.7 (−4.0–66)	0.50
AI tAUC (% · 180 min)	4839 ± 360 (2880.0–7080.0)	4140 ± 787 (−2190.0–10,530.0)	0.43
Fasted plasma glucose (mg/dL)	103.3 ± 2.8 (91.3–122.0)	100.2 ± 2.6 (86.0–114.0)	0.42
2‐h plasma glucose (mg/dL)	148.8 ± 10.1 (99.5–217.0)	130.3 ± 10.6 (77.3–185.0)	0.22
Glucose tAUC_180_ (mg/dL·min)	26,002.2 ± 1424.4 (18,415.5–36,090.0)	23,998.3 ± 1523.6 (15,413.3–34,207.5)	0.35
Triglycerides (mg/dL)	131.7 ± 19.0 (56.0–271.0)	115.6 ± 23.9 (57–386.0)	0.61
LDL (mg/dL)	128.8 ± 14.7 (67.0–259.0)	116.8 ± 6.3 (86.0–156.0)	0.44
HDL (mg/dL)	51.6 ± 4.5 (40.0–95.0)	50.1 ± 3.6 (31.0–77.0)	0.79
Total cholesterol (mg/dL)	202.2 ± 16.8 (134.0–346.0)	186.1 ± 8.0 (144.0–239.0)	0.39
White blood cell (k/*μ*L)	5.6 ± 0.4 (3.75–8.30)	5.8 ± 0.3 (4.23–7.80)	0.66

Data presented are mean ± SEM. BMI, body mass index; tAUC, total area under the curve; LDL, low‐density lipoprotein; HDL, high‐density lipoprotein; MAP, mean arterial pressure; AI, augmentation index.

### Metabolic control

Subjects were instructed to refrain from strenuous exercise, caffeine, or alcohol consumption for 48‐h prior to testing. Subjects were also asked to refrain from taking any medications or dietary supplements 24‐h prior to reporting to the Clinical Research Unit. Subjects recorded habitual dietary intake 3‐day before testing to confirm mixed meal consumption and were then instructed to consume 250 g/day of carbohydrates on the day before testing. There was no difference between habitual diet and food consumed prior to testing, so the 4 days were averaged for analysis.

### Cardiorespiratory fitness

VO_2_peak was determined using a continuous progressive exercise test on a cycle ergometer with indirect calorimetry (Carefusion, Vmax CART, Yorba Linda, CA). Heart rate and blood pressure were obtained at rest and heart rate was continuously monitored using a 12‐lead EKG. The power output was increased by 25 watts every 2 min until the subject met volitional exhaustion, RER was >1.1 and the cadence dropped below 60 rpm.

### Body composition

Following an approximate 4‐h fast, body weight was measured to the nearest 0.01 kg on a digital scale with minimal clothing and without shoes. Subjects were instructed to wipe their hands and feet with an anti‐bacterial cloth prior to measurement to enhance electrical conductivity. Percent body fat and fat‐free mass were measured using InBody 770 Body Composition Analyzer (InBody CO, Cerritos, CA) (Faria et al. 2014). Waist circumference was measured 2 cm above the umbilicus (Malin et al. 2012).

### Oral glucose tolerance test (OGTT) and arterial stiffness

After an approximate 10–12‐h fast, subjects reported to the Clinical Research Unit. Subjects were then instructed to lay supine undisturbed for at least 5 min to determine resting heart rate and blood pressure, which was averaged over three measurements for data analysis. Additionally, pulse pressure (defined as systolic‐diastolic blood pressure) and mean arterial pressure [((2*diastolic) + systolic)/3] were calculated. Blood samples were drawn from an antecubital vein after placement of an indwelling catheter. Blood lipids and white blood cells were analyzed using enzymatic colorimetric‐based assays via our University Medical Laboratories. A 75 g OGTT was then performed to assess glucose tolerance by determining plasma glucose every 30 min up to 120 min and then 180 min. Plasma glucose samples were analyzed using the YSI 2300 StatPlus Glucose Analyzer System (Yellow Springs, OH). Augmentation index (AI) was measured using the SphygmoCor^®^ system, (AtCor Medical, Itasca, IL) for determination of arterial stiffness at minutes 0, 60, 120, and 180 min. AI was corrected to a heart rate of 75 bpm using the manufacturer's software. Total area under the curve (tAUC) was calculated using the trapezoidal model.

### EV preparation

Fresh blood prior to the OGTT was collected in citrate vacutainers and processed within 120 min of collection for the measurement of platelet (CD31^+^/CD41^+^), leukocyte (CD45^+^/CD41^−^), endothelial (CD105 and CD31^+^/CD41^−^) EVs as described previously by Erdbrügger and Lannigan (2016). Annexin V (AV) was used as a membrane dye. Platelet poor plasma was obtained by centrifugation (Sovall RC 5B Plus Centrifuge: Rotor SS‐34 Fixed Angle Rotor) at 5000 g at room temperature for 15 min. An EV pellet was obtained from platelet poor plasma by a second centrifugation spin (Centrifuge: 524/5424 R‐Rotor FA‐45‐24‐11) at 17,000 g for 10 min, which was then washed with PBS + 0.5% BSA, repelleted, and resuspended with 1× Annexin V buffer (1× AVb) (BD Parmingen, San Diego, CA). 100 *μ*L of each washed EVs were split into seven microfuge tubes. For the samples, 20 *μ*L of the antibody mix was added to one tube, while nothing was added to the second tube. For the controls, 20 *μ*L of the antibody mix was added to 100 *μ*L of the AV buffer. For compensation controls, 1 *μ*L of each individual fluorescence (FITC (Annexin V, Biolegend^®^), PE (CD105, Biolegend^®^), PacBlue (CD41, Biolegend^®^), BV510 (CD 45, Biolegend^®^), AF647 (CD31, Biolegend^®^) was added to one tube. The samples were then vortexed three times, for 5 sec and then incubated 45–60 min in the dark at room temperature. After this time period, 1 mL of 1× AV buffer was added to all seven tubes. The pellet EVs were spun for 10 min at 17,000***g*** at room temperature. The supernatant was removed, leaving approximately 10–20 *μ*L in the tube. Between 30 and 40 *μ*L of the 1× AV Buffer was added to all seven tubes again, leaving approximately 50 *μ*L of solution in each tube. The samples were vortexed three times for 5 sec. Upon completion, EVs in the sample were concentrated two fold.

### EV imaging

**Figure 1:**
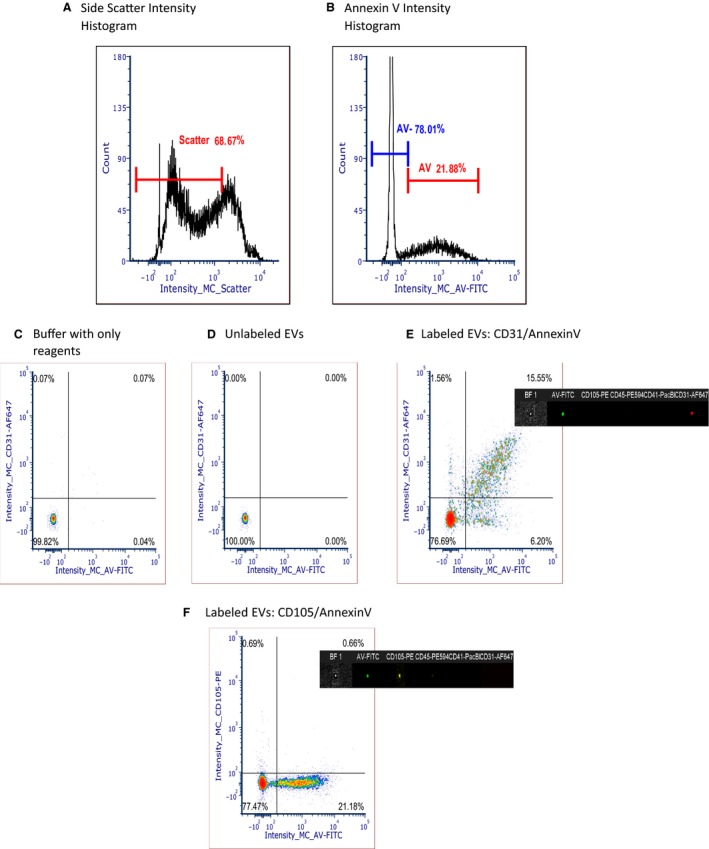
EV Phenotyping with Imaging Flow Cytometry. Gating strategy based on low scatter (A) and Annexin V intensity positivity (B) on intensity histogram according to our previous published methods, (C) and (D) Controls: Buffer with only reagents, no EVs (C) unlabeled EVs without reagents (D). Example of dot plots of EV labeling: (E): CD31/Annexin V density plot and (F) CD105/Annexin V density plot.

Imaging flow cytometry was used to isolate and determine the source and count of the EVs as described previously by our group (Erdbrügger et al. 2014; Erdbrügger and Lannigan 2016). The flow cytometer ImageStream^®X^ MKII (Amnis, Seattle, WA) (ISX) was utilized, as it combines the capabilities of conventional flow cytometry (FCM), with high‐resolution imaging at the single cell level to accurately depict EV subtypes. All lasers of the ImageStream^®X^ MK II are set to full power, including the scatter laser. Magnification was set to 60× and core size reduced to 7 *μ*m. Samples were loaded and acquired for 2 min (or specific fixed time for all samples). Acquisition gates were set on low scatter signals that are 2–3 decades lower than speed beads (Fig. 1A and B). We used several controls, including compensation controls as single stained EV samples, Buffer only controls (collected for 2 min after filtering with a 0.1 *μ*m filter, Buffer plus reagents control (to rule out that antibodies by itself do not mimic appearance of EVs e.g. by aggregation (Fig. 1C) and an unlabeled EV control sample (to establish the gating of subpopulations (Fig. 1D)). EV counts were measured by a volumetric method provided by the software of ISX. The acquired raw data were then analyzed using IDEAS software. FCS Express6 DeNovo TM software was then used to create the histogram and dotplots.

### Cryo‐electron microscopy to image EVs

**Figure 2:**
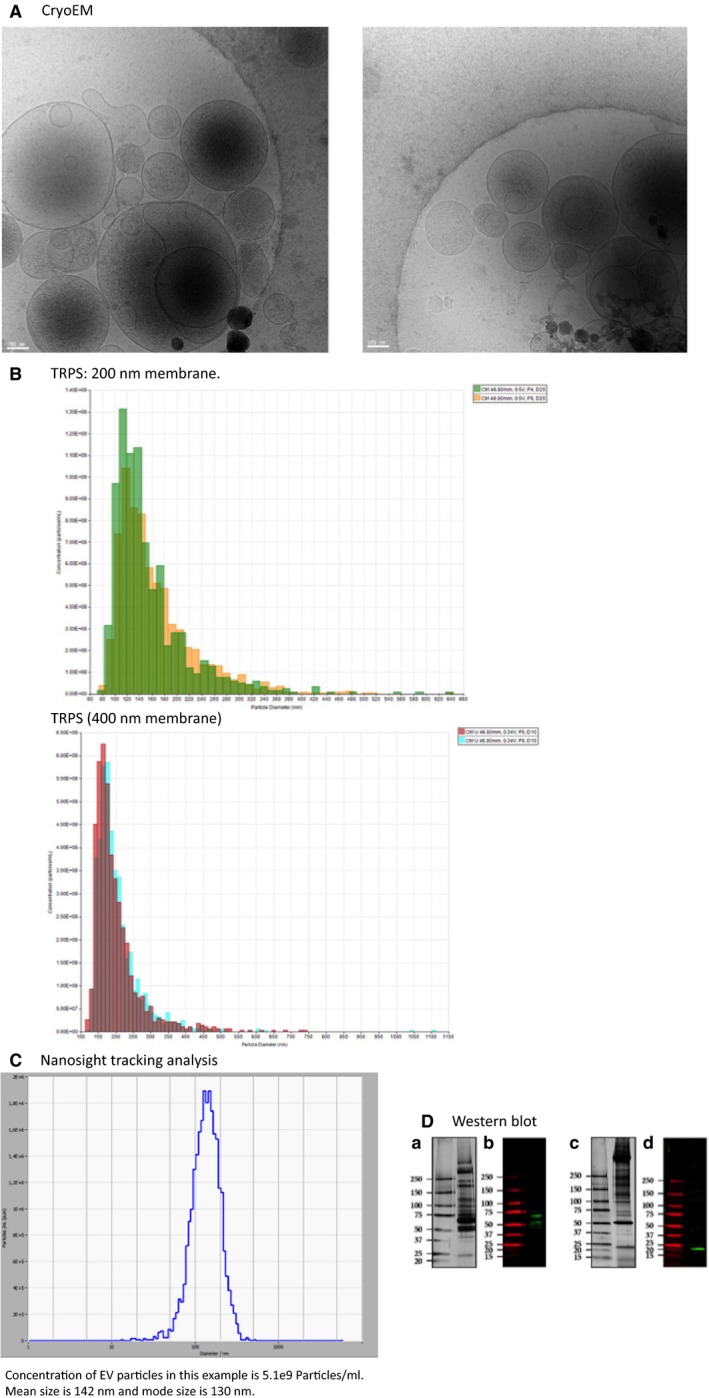
Characterization of EV size, concentration, and morphology. Cryo‐electron microscopy images of EVs of different sizes (<100 nm to 1000 nm) (A); Tunable resistive pulse sensing (TRPS, qNano^®^ by Izon, using 200 nm and 400 nm with 2 pressures). Concentration of EV particles in this example using a 200 nm pore size for qNANO with two pressures at 4A and 8A is 5.7e9 Particles/ml. Mean size is 161 nm and mode size is 116 nm taking average of both pressure measurement. Concentration of EV particles in the same example using a 400 nm pore size for qNANO with 2 pressures at 5A and 8A is 4.5e9 Particles/mL. Mean size is 203 nm and mode size is 170 nm taking average of both pressure measurements (B). Particle tracking of EVs with Nanosight tracking analysis (Zetaview^®^ by Particle Metrix). Concentration of EV particles in this example is 5.1e9 particles/mL. Mean size is 142 nm and mode size is 130 nm (C); Western blotting of vesicle proteins. *A* (protein pattern) and *B* (Western blotting) show vesicle protein TSG101 in reducing condition and *C* (protein pattern) and *D* (Western blotting) show vesicle protein CD9 in nonreducing condition at expected band length (D).

Purified samples were verified by standard methods for cryo‐electron microscopy (cryoEM) to determine EV morphology (Yeager et al. 1994). In brief, an aliquot (3 mL) was applied to a glow‐discharged, perforated carboncoated grid (2/2‐4C C‐flats), blotted with filter paper, andrapidly plunged into liquid ethane. Low‐dose images were recorded at a magnification of 29,0003 on a FEI Tecnai F20 Twin transmission electron microscope operating at 120 kV, with a nominal underfocus ranging from 3.5 to 5 mm and a pixel size of 0.388 nm at the specimen level. All images were recorded with a Gatan 4K 3 4K pixel CCD camera. The grids were stored in liquid nitrogen, and then maintained in the microscope at 2180_C using a Gatan 626 cryo‐stage. Samples were also prepared for scanning electron microscopy (Fig. 2A).

### EV size detection

Tunable Resistive Pulse Sensing (TRPS) was performed with a gold qNano instrument (Izon Ltd) mounting a polyurethane nanopore membrane NP200 (range 85–500 nm) and NP400 (range 125–1100 nm) (Izon Ltd). Multi pressure at 4, 5, and 8 mBar, respectively, was applied to determine the particle concentration. Electrolyte solution was made of PBS supplemented with 0.03% (v/v) Tween‐20 filtered with Minisart^®^ high flow hydrophilic 0.1 *μ*m syringe filter (Sartorious). Current pulse signals were collected using Izon Control Suite 3.2 software (Izon Ltd). EV pellet after differential centrifugation was solubilized in 50 *μ*l of filtered electrolyte solution. Polystyrene particle standards (SPK200B and CPC400B; Izon Ltd.) were employed for calibration. Both uEVs pellet and particle standards were measured with a minimum of 1000 blockades (Fig. 2B). Nanosight Tracking Analysis (NTA) was carried out using the Zetaview PMX 110 multiple parameter particle tracking analyzer (particle metrix, Meerbusch, Germany) in size mode using Zetaview software version 8.02.28. Plasma samples were diluted in 1× pbs to the working range of the system. The system was calibrated using 105 nm polystyrene beads and then plasma vesicle profiles were recorded and analyzed at 11 camera positions with a 2 sec video length, a camera frame rate of 30 fps and a temperature of 21°C (Fig. 2C).

### Western blotting

Protein quantification of EVs was performed by Coomassie microassays. EV pellets were solubilized in 40 *μ*L of solubilization buffer made of 5% (w/v) sodium dodecyl sulfate (SDS), 40 mmol/L Tris‐HCl pH 6.8, 0.5 mmol/L ethylenediaminetetraacetic acid (EDTA), 20% (v/v) glycerol without and with 50 mmol/L dithiothreitol (DTT), respectively. Samples were denaturated overnight at room temperature. Proteins were separated by hand cast SDS‐PAGE gradient gels (Resolving gel T = 5–20% (w/v); C = 2.6%; Stacking gel T = 3.5% (w/v); C = 2.6%) in 25 mmol/L Tris, 192 mmol/L glycine and 0.1% (w/v) SDS buffer and either stained with silver staining or transferred onto a 0.45 *μ*m nitrocellulose membrane (Amersham™ Protan™ 0.45 *μ*m NC, GE Healthcare) in a wet transfer system buffer made of 25 mmol/L Tris, 192 mmol/L glycine and 20% (v/v) methanol. Nitrocellulose membranes were saturated with Odyssey blocking buffer (LI‐COR Biosciences) and incubated in polyconal rabbit anti TSG101 (Sigma, T5701‐200UL) and monoclonal mouse anti CD9 (HansaBiomed; HBM‐CD9‐100) overnight at room temperature (RT = 23–24°C) in an Odyssey blocking buffer diluted 1:1 with PBS and 0.15% (v/v) Tween‐20 at concentration of 1.0 *μ*g/mL. After 3 × 10 min washes in PBS‐Tween (0.15%, v/v), membranes were incubated with anti rabbit anti mouse dye‐coupled secondary antibody 0.1 *μ*g/mL (LI‐COR Biosciences) in an Odyssey blocking solution diluted at 1:1 with PBS and 0.15% (v/v) Tween‐20; 1‐h at RT. Acquisition of the fluorescent signal was performed by Odyssey infrared imaging system with resolution set at 169 *μ*m (LI‐COR Biosciences) (Fig. 2D). We have submitted all relevant data to the EV‐Track knowledgebase (EV‐TRACK ID: EV180013) (Van Deun et al. 2017).

### Statistical analysis

Data were analyzed using SPSS v24 (Armonk, NY). Data were log‐transformed in the event normal distribution was not achieved. VPF and PF group differences were compared using independent, two‐tailed *t*‐tests. Sex differences were compared utilizing the Fischer exact test. Given that age and body fat percentage were statistically significant between groups, we conducted an analyses of covariance to test independent effects of fitness on EVs. We also conducted a subanalysis including women only to test sex effects based on current mixed literature (Toth et al. 2007; Gustafson et al. 2015; Lansford et al. 2016). Pearson correlation was used to assess associations. Statistical significance was set at *P *≤* *0.05. Adjusted means are presented throughout the manuscript and data are reported as mean ± SEM.

## Results

### Subject demographics

Body weight, waist circumference, blood pressure, plasma lipids, and circulating glucose were similar between VPF and PF (Table 1). There was no significant difference between groups in total calories (VPF: 2020.8 ± 165.2 vs. PF: 2343.9 ± 188.0, *P *=* *0.21), carbohydrates (VPF: 48.9 ± 3.7 vs. PF: 48.5 ± 2.2%, *P *=* *0.93), protein (VPF: 16.7 ± 1.4 vs. PF:16.3 ± 0.8%, *P *=* *0.80), or fat (VPF: 36.7 ± 2.7 vs. PF: 35.4 ± 2.1%, *P *=* *0.70). However, individuals with VPF by study design had a lower VO_2_peak than those with PF (*P *<* *0.01). Subjects with VPF also had more women (*P *=* *0.09), higher age (*P *=* *0.007), and increased body fat (*P *<* *0.001; Table 1). All but two women (both in PF) were postmenopausal.

### Characterization of EVs

We report a heterogeneous size of EVs (Fig. 2A), ranging from 80 to 600 nm in size. Importantly, the size of EVs were similar, regardless of measurement by TPRS or NTA (Fig. 2B and C). Using two different membrane sizes of 200 and 400 nm for the TRPS analysis, with two different pressures, the mean size of EVs was 182 nm and mode size was 142 nm, whereas the mean size of NTA was 168 nm and mode size was 138 nm. We also verified by Western blot, that our method was appropriate for detecting vesicles by comparing two established proteins, namely tetraspanin CD9 and ESCRT, an endosomal sorting complex required for transport component marker TSG101. While CD9 was detected at its own molecular weight at 20–25 kDa, TSG101 presented a set of bands at molecular weight higher than the nominal one at 46 kDa which can be attributed to multiple ubiquitination site (Tal, a Tsg101‐specific E3 ubiquitin ligase, regulates receptor endocytosis and retrovirus budding) and/or ISGylation (Fig. 2D).

### EV subtype phenotyping

**Figure 3:**
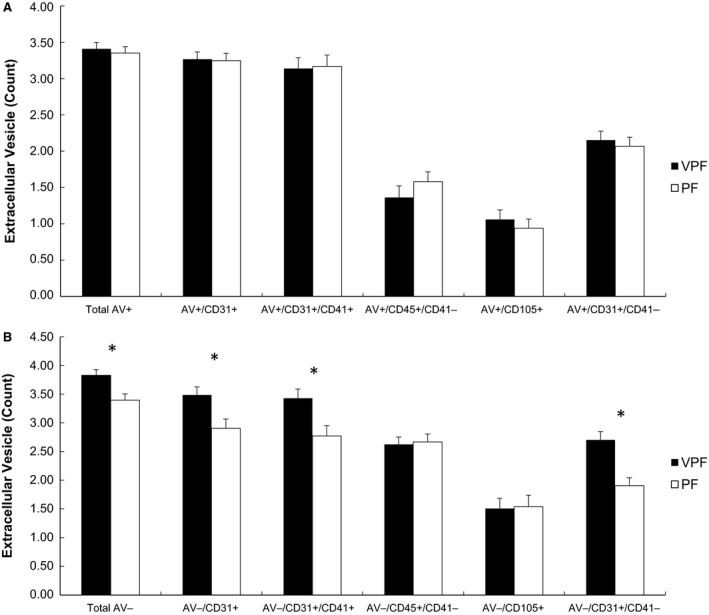
Correlation between CD31^+^/CD41^−^ endothelial EVs and pulse pressure (A) and CD105 + endothelial EVs with 2‐h glucose (B). EV data were log‐transformed.

Total AV^−^ EVs were significantly elevated in people with VPF compared with PF (*P *=* *0.02). In addition, individuals with VPF had higher platelet EVs (AV^−^/CD31^+^/CD41^+^, *P *=* *0.03) and endothelial EVs (AV^−^/CD31^+^/CD41‐, *P *=* *0.002) when compared with those with PF independent of age and body fat percentage (Fig. 3A). However, there was no significant difference between VPF and PF in leukocyte‐derived AV^−^/CD45^+^/CD41‐ EVs (*P *=* *0.70) or the endothelial EV AV^−^/CD105^+^ *(P *=* *0.28). There were no significant group differences in any AV^+^ EV subtype when stratified based on fitness level (Fig. 3B), however, the endothelial‐derived EV AV^+^/CD31^+^/CD41‐trended to be higher in the VPF group (*P *=* *0.07; Fig. 3B). Subanalysis of women verified higher total AV^−^ EVs in VPF than PF (3.8 ± 0.09 vs. 3.4 ± 0.12, *P *=* *0.04). Moreover, AV^−^/CD31^+^ (2.96 ± 0.14 vs. 3.5 ± 0.10, *P *=* *0.01), AV^−^/CD31^+^/CD41^+^ platelet (2.8 ± 0.2 vs. 3.4 ± 0.1, *P *=* *0.006), and AV^−^/CD31^+^/CD41^+^ endothelial EVs (1.9 ± 0.2 vs. 2.7 ± 0.1, *P *=* *0.005) were also higher in VPF women independent of age and body fat.

### Correlations

**Figure 4:**
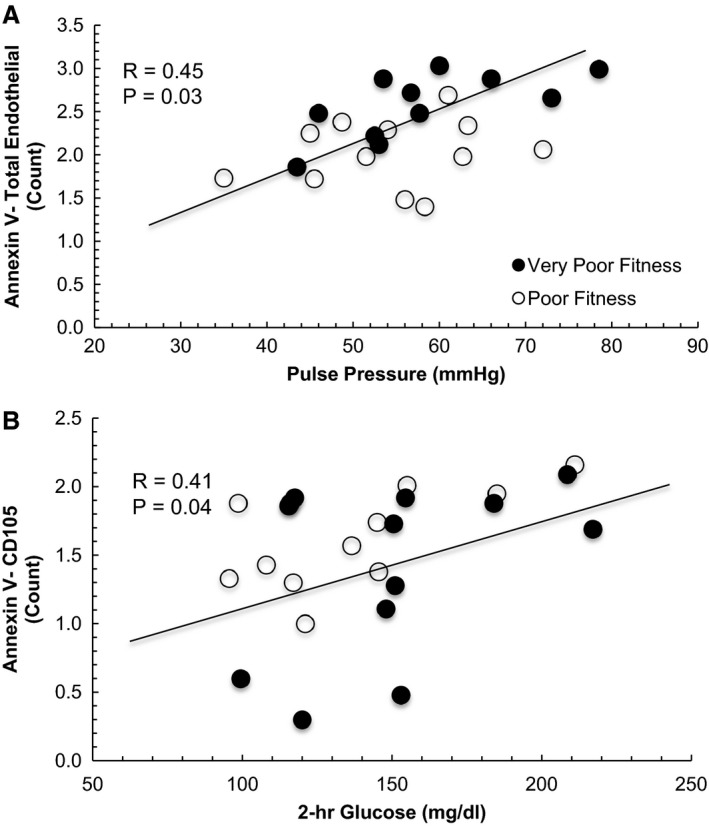
Comparison of Annexin V^+^ (A) and Annexin V^−^ EV subtypes (B) in obese individuals with very poor fitness (VPF) and poor fitness (PF). EV data were log‐transformed. Subtypes: CD41 (platelets), CD105 (S‐endoglin, endothelial), CD31 (PECAM, platelet endothelial cell adhesion molecule). Age and body fat were included as covariates.

Low VO_2_peak was significantly associated with higher AV^−^/CD31^+^ (*r* = −0.52, *P *=* *0.009), AV^−^/CD41^+^ (*r* = −0.57, *P *=* *0.003), AV^−^/CD31^+^/CD41^+^ platelet EVs (*r* = −0.55, *P *=* *0.005), and AV/CD31^+^/CD41^−^ endothelial EVs (*r* = −0.56, *P *=* *0.006). Elevated AV^−^/ CD31^+^/CD41^−^ endothelial EVs also correlated with increased pulse pressure (*r* = 0.45, *P *=* *0.03; Fig. 4A). Moreover, endothelial EV AV^−^/CD105^+^ was positively related to 120‐min glucose (*r* = 0.41, *P *=* *0.04; Fig. 4B) following the OGTT. High AV^−^/CD45^+^/CD41^−^ leukocyte EVs tended to correlate with decreased HDL cholesterol (*r* = −0.43, *P *=* *0.06) and increased arterial stiffness (*r* = 0.40, *P *=* *0.06). Increased arterial stiffness (measured as tAUC_180_) was also associated with decreased fitness (*r* = −0.47, *P *=* *0.02). No other significant correlations were found between AV^−^ EV subtypes and body fat, BMI, blood pressure, LDL, total cholesterol or triglycerides (data not shown).

## Discussion

The major finding of this study using detailed characterization of EVs is that individuals with VPF have elevated levels of both AV^−^ platelet and endothelial‐derived EVs when compared with individuals with PF (Fig. 4A). These findings may be of clinical relevance since elevated platelet and endothelial EVs are in concordance with increased T2D and CVD risk (Nomura 2009; Amabile et al. 2010). Subtle reductions in cardiorespiratory fitness by just 1‐MET is related to increased mortality risk by 13% (Kodama et al. 2009), suggesting that even slight improvements in fitness may be cardioprotective. To date, the mechanism by which subtle differences in fitness promote this health benefit remains largely unknown. Van Craenenbroeck et al. (2015) previously suggested that baseline endothelial EVs were inversely correlated with increases in VO_2_peak and endothelial function following a 3‐month exercise intervention in older adults with coronary artery disease and poor fitness. In line with these observations, Navasiolava et al. (2010) reported that only 7 days of physical inactivity raised endothelial‐derived EVs in association with reduced basal flow and endothelium‐dependent vasodilation in healthy male adults. Taken together, our work extends these findings by showing that subtle elevations in aerobic fitness may be categorized by lower EV counts of platelet and endothelial, but not leukocyte, and confer cardiometabolic health in obese individuals.

There are several possible roles by which fitness may be related to endothelial and/or platelet EV subtypes in these obese individuals. Previous work from our group has shown subcutaneous and abdominal visceral fat to be elevated in obese women with PF (Irving et al. 2008). This is potentially problematic because obese adults have elevated fat‐derived hormones that promote inflammation and reduce insulin sensitivity and cardiometabolic health (Malin et al. 2013a). Since inflammation is a purported mechanism involved in the regulation of endothelial EV release (Dignat‐George and Boulanger 2011), it would be reasonable to expect that differences in body fat could relate to elevations in endothelial EV subtypes. However, although people with VPF had higher body fat when measured with bioelectrical impedance when compared with PF, our results indicate that AV^−^ platelet (CD31^+^/CD41^+^) and endothelial (CD31^+^/CD41^−^) EVs remained significantly elevated in people with VPF when compared with PF individuals after controlling for body fat. These data suggest that fitness is related to differences in EVs independent of total body fat.

Inflammatory processes may drive atherosclerosis and CVD risk independent of total adiposity and help explain the impact of fitness on platelets EVs (Hansson 2005). Although previous literature has shown VO_2_peak to be inversely related to CRP and inflammation (LaMonte et al. 2002; Lavie et al. 2011), no previous work has examined the relationship of fitness with platelet EVs and inflammation. Our results therefore fill this knowledge gap, as we observed significantly lower platelet EVs in people with PF compared with VPF. The clinical relevance of these elevated platelet EVs, however, remain unclear as we did not find any significant correlation with platelet EVs. This may be due to the underlying pathophysiology of our specific clinical population, as even WBCs, a clinical marker of inflammation was unrelated to platelet EVs. Thus, future studies should consider examining more specific measures of inflammation, including CRP, IL‐6, or TNF*α* since they have been suggested to impact EV release (Nomura et al. 2000).

Another possible factor related to subtle differences in fitness contributing to lower EV levels may relate to vascular function. Indeed, circulating EVs play an important physiologic role in vascular physiology (Dignat‐George and Boulanger 2011) and elevated endothelial EVs correlate with reduced endothelium‐dependent vasorelaxation (Werner et al. 2006) and flow‐mediated dilation (Esposito et al. 2004), as well as increased arterial stiffness (Wang et al. 2009). Interestingly, we observed that elevated VO_2_peak was significantly correlated with lower arterial stiffness. This finding is consistent with others showing that cardiorespiratory fitness is associated with improved endothelial function and lower blood pressure (Niebauer and Cooke 1996). Thus, it would be expected that elevated levels of circulating endothelial EVs would correlate with increased blood pressure through a fitness‐related mechanism. Interestingly, we report that elevated AV^−^ endothelial EVs (CD31^+^/CD41^−^) correlated with increased pulse pressure (Fig. 4A), suggesting that endothelial EVs may play a role in blood pressure and CVD risk (Preston et al. 2003). Although this study was not designed to test how fitness modifies endothelial EVs, we speculate that the higher levels of shear stress with physical activity in people with PF, compared with VPF, may counteract EV release (Boulanger et al. 2007; Thosar et al. 2012). Indeed, our data are consistent with in vitro work demonstrating that endothelial EVs promote vascular dysfunction by impairments in nitric oxide release and/or increased apoptosis of endothelial progenitor cells (Pirro et al. 2006).

Cardiorespiratory fitness contributes to improved insulin sensitivity that in part explains lower blood glucose levels and type 2 diabetes risk (Malin et al. 2012). Recent work by Burger et al. (2017) assessed the effect of high glucose exposure to HUVEC cells on endothelial EVs and reported that hyperglycemia increased endothelial EV count, promoted greater procoagulant activity, elevated reactive oxygen species, and blunted endothelial relaxation. This is line with previous work that suggested high glucose conditions increased NADPH oxidase activity in endothelial EVs, thereby promoting vascular inflammation (Jansen et al. 2013). Consistent with these recent in vitro studies, we report that high 2‐h plasma glucose concentrations were directly correlated with endothelial EV AV^−^/CD105 (Fig. 4B). These findings suggest that hyperglycemia may be an important modifier of vascular function that contributes to fitness‐related adaptation that lower risk of type 2 diabetes. Interestingly, this is consistent with recent work showing that endothelial EVs are higher in people with prediabetes when compared with adults with normal glucose tolerance (Giannella et al. 2017). Whether prospective exercise interventions in people with prediabetes can alter EVs in relation to vascular adaptation remains to be determined.

Leukocyte EVs were not associated with fitness in this study, although they did tend to correlate with both increased arterial stiffness and decreased HDL cholesterol. These observations suggest that leukocyte EVs may have clinical relevance in obese adults. In fact, high leukocyte‐derived EVs were previously related to higher inflammation (indicated by hs‐CRP) in people with metabolic syndrome when compared with healthy counterparts (Chironi et al. 2006). We speculate that we did not see a difference in leukocyte EVs in this study due to a lack of difference in WBC counts between groups. Nonetheless, the interplay between leukocyte EVs and arterial stiffness and HDL cholesterol may be physiologically meaningful and additional studies are needed to definitively determine the impact of fitness on leukocyte EVs to understand their clinical relevance.

This study has several limitations that may impact our interpretation. This was a cross‐sectional and correlation analysis does not imply causation. Additionally, this was a relatively small sample size and we cannot generalize these findings across race, which has been documented to relate to EVs (Brown et al. 2011). There were males in the PF group whereas the VPF group was entirely female, thereby raising questions on the role of sex explaining differences in EV subtypes between groups (Toth et al. 2007; Lansford et al. 2016). Although sex differences have been reported in EVs, this difference is not consistently reported across all EV subtypes (LaMonte et al. 2002; Pirro et al. 2006). Our subanalysis including females only demonstrate that there are still significant fitness‐related EV differences. Thus, sex is unlikely to influence our present findings, however, we are likely underpowered to definitively determine if sex differences exist and future research is warranted. We also observed that individuals with VPF were older than the PF participants, as well as had a higher percentage of body fat that could collectively explain why higher EV levels were observed between these cohorts. However, after including both of these covariates in our ANOVA model, we still saw that those individuals with lower fitness levels had higher levels of EVs when compared with individuals with slightly higher levels of fitness. Additionally, no direct relationships were observed between any AV^−^ EV subtypes and age or body fat % (data not shown), suggesting that fitness is potentially an important modifier of EVs. Diet is another factor that may impact our results (Ferreira et al. 2004; Tushuizen et al. 2006; Jansen et al. 2013; Bruyndonckx et al. 2015). Although we did not strictly control for macronutrient intake proceeding measures in this study, there no statistical difference in caloric intake between groups, suggesting that diet is unlikely to influence our fitness mediated results. Nevertheless, a major strength of this study is the use of fresh blood samples combined with imaging flow cytometry to more accurately assess EV counts (van Ierssel et al. 2010) across subtypes (Erdbrügger and Lannigan 2016). In fact, our approach used EV‐Track (http://www.evtrack.org) reporting and highlights not only EV origin (flow cytometry) but also EV size (TRPS and NTA), morphology (cryo EM), and proteins (Western blotting). These EV‐Track guidelines are a recent attempt to improve transparency of methodology within the field of extracellular vesicles, as great heterogeneity of both isolation and characterization of exists (Van Deun et al. 2017). As we are in accordance with these guidelines, these data add to the literature and strengthen our claim that EVs have clinical and aerobic fitness‐related relevance. Indeed, this study is the first to report significant findings between various clinical outcomes and AV^−^ EVs, as previous literature has reported mostly about AV^+^ EV subtypes, which is likely due to the limitation of conventional flow cytometry capabilities in measuring smaller EV sizes of about 200–400 nmol/L depending on the flowcytometer used (Erdbrügger et al. 2014; Erdbrügger and Lannigan 2016).

In conclusion, VPF is associated with higher AV^−^, endothelial and platelet EVs in obese adults, suggesting that subtle differences in fitness may induce cardioprotection, in part, through an EV‐subtype‐related mechanism. Moreover, we identified that EVs were significantly correlated with lower arterial stiffness and blood glucose, thereby highlighting potential connections with development of hypertension and type 2 diabetes. Indeed, these results support the need to examine both AV^+/−^ EVs, as well as smaller vesicles, such as exosomes (40–100 nm), in future clinical work to better understand the etiology of cardiometabolic disease. Overall, these fitness‐related findings suggest that vascular and metabolic adaptations to physical activity/exercise elicit cell‐specific EV responses, and future work is warranted to elucidate the mechanism by which EVs‐induce cardiometabolic health differences before after exercise interventions to optimize prevention and/or treatment of chronic disease.

## Conflict of Interest

Authors have nothing to disclose.
